# Video-assisted direct leadless pacemaker implantation during open heart valve surgery

**DOI:** 10.1016/j.hrcr.2024.04.012

**Published:** 2024-05-03

**Authors:** Justin Arunthamakun, Giorgio Zanotti, Benzy J. Padanilam

**Affiliations:** ∗ Department of Electrophysiology, Ascension St. Vincent Hospital, Indianapolis, Indiana; † Department of Cardiothoracic Surgery, Ascension St. Vincent Hospital, Indianapolis, Indiana

**Keywords:** Heart block, Leadless pacemaker, Open heart valve surgery, Valve replacement, Video-assisted

**Key Teaching Points**

• Direct intraoperative Micra (Medtronic, Minneapolis, MN) leadless pacemaker implantation is feasible.
• Anchoring of tines in trabeculations during tug test can be confirmed with video visualization.
• Direct visualization of the right ventricular septum minimizes the risk of cardiac perforation.
• Cardioplegia limits testing of device parameters immediately after implantation.

## Introduction

Leadless pacemaker implantation is generally performed transvenously from the femoral vein or internal jugular vein.^1^^,^^2^ Fluoroscopy guides the catheter to the right ventricular (RV) septum, minimizing risk of cardiac perforation related to the implantation. While several fluoroscopic techniques are described to identify the RV septum, none are definitive, and cardiac perforation can be a devastating complication because of the large size of the sheath and device.^3^, ^4^, ^5^ During open heart surgery the RV septum and trabeculations may be directly visualized, avoiding the risk of cardiac perforation. We present 2 cases of intraoperative videoscope-assisted Micra (Medtronic, Minneapolis, MN) leadless pacemaker implantation with description of a new methodology to confirm engagement of pacemaker tines in RV trabeculations without the use of fluoroscopy.

## Case report

### Patient 1

An 88-year-old woman with cardiac amyloidosis and severe tricuspid and mitral regurgitation was admitted for a minimally invasive port-access mitral and tricuspid valve repair. Prior to her surgery, she was noted to have long-standing persistent atrial fibrillation with symptomatic slow ventricular rates. She was recommended a leadless pacemaker implantation to avoid placing a ventricular lead across a repaired tricuspid valve. It was felt that an intraoperative leadless pacemaker implantation would be most appropriate owing to her comorbidities and risk factors.

**Figure 1:**
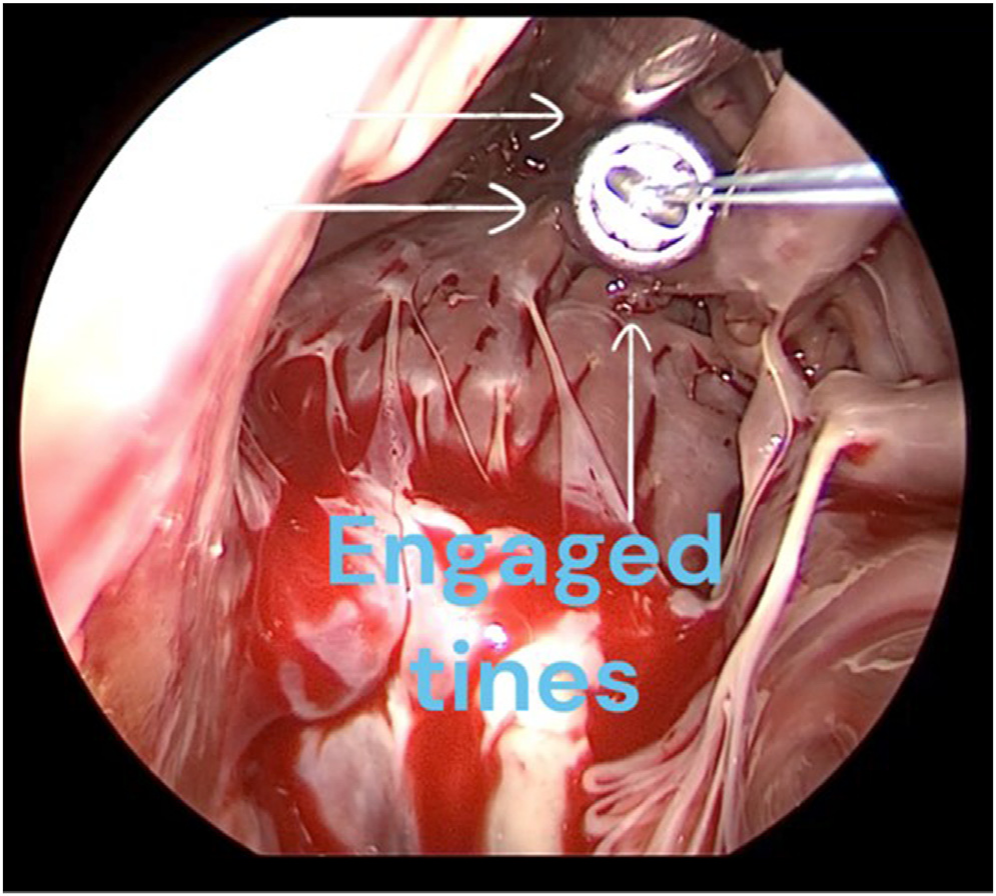
Intraoperative videoscope image showing engaged nitinol tines of the Micra leadless pacemaker (Medtronic, Minneapolis, MN) during the tug test. The white arrows point to the 3 tines that malformed right ventricular myocardial trabeculations, showing evidence of engagement. See Supplemental Video 1 for better perspective.

**Figure 2:**
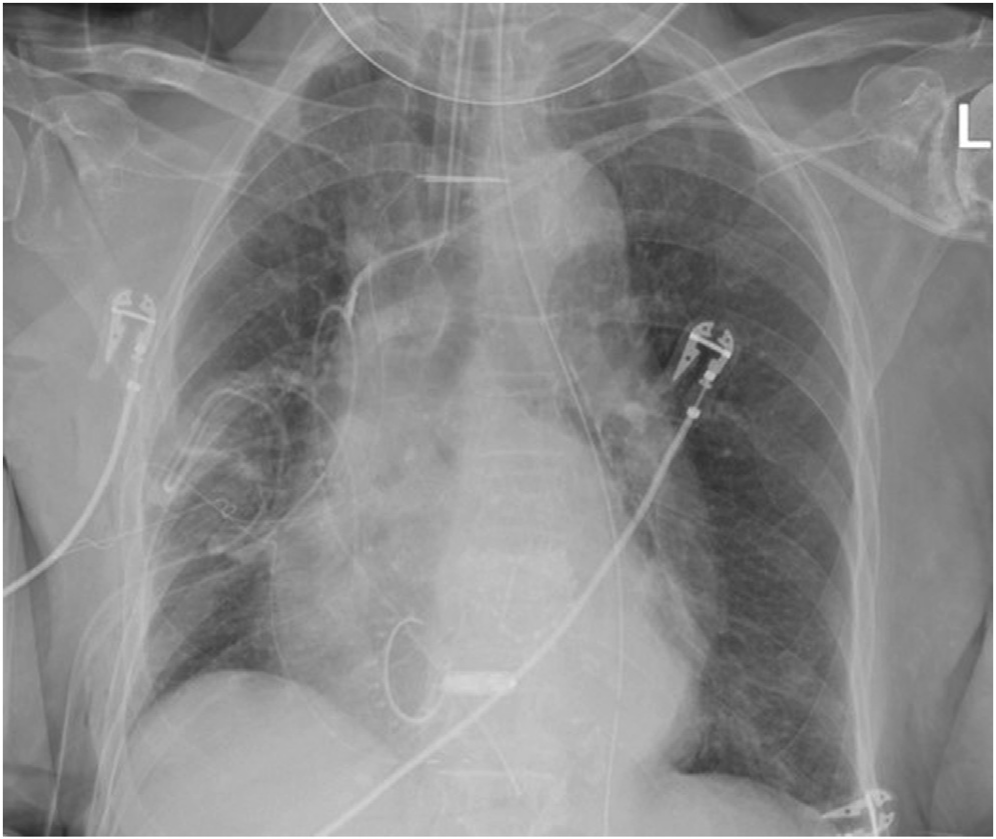
Patient 1 chest radiography on postoperative day 1, which demonstrates a well-positioned leadless pacemaker and tricuspid and mitral rings.

Lateral port access was obtained, and the patient was placed on cardiopulmonary bypass and cardioplegia. The mitral annulus was repaired with a 28 mm Memo 3D ring (Corcym, Saluggia, Italy). The right atrium and the right ventricle were exposed for tricuspid valve repair. The Micra delivery apparatus was introduced into the right ventricle directly through the open right atrium and tricuspid valve. The surgeon directed the delivery sheath tip on the RV septum in an area of trabeculations. The surgeon held a forward force on the sheath and the Micra VR pacemaker was advanced using the standard technique. Next, the engagement of the flex-fix nitinol tines was tested with the tug test. Video evidence of tine engagement was obtained with 3 different tines showing retraction of trabeculations during tug test (Supplemental Video 1 and Figure 1). As the tugging pulled the pacemaker back, the operator could feel the tension on the tethers and the tines could be seen via videoscope to malform the trabeculations. Pacemaker sensing and pacing threshold and impedance could not be performed immediately, as the patient was under cardioplegia. The tethers were cut and the sheath removed. The tricuspid valve was repaired with a 28 mm MC3 tricuspid valve ring (Edwards, Irvine, CA). Once cardiac activity was restarted, testing of the device showed an R-wave sensing of 7.5 mV, lead impedance of 410 Ω, and pacing threshold of 1.88 V @ 0.4 ms. The device was programmed to VVIR 80 beats per minute (bpm) with ventricular output of 3 V @ 0.5 ms. Final intraoperative transesophageal echocardiogram showed a mid-septal position of the Micra leadless pacemaker and appropriate function of both prosthetic mitral and tricuspid valves. The day following her surgery, the device continued to function properly with R waves of 2.4 mV, 390 Ω impedance, and threshold of 1.88 V @ 0.4 ms. Figure 2 shows her postoperative chest radiograph, which showed a well-positioned leadless pacemaker and surgical tricuspid and mitral rings. She was discharged from the hospital on postoperative day 6. On her 3-week follow-up, the pacemaker showed a higher pacing threshold of 3.1 V @ 0.4 ms and stable device parameters with R-wave sensing of 4.1 mV, and 420 Ω impedance. Her device parameter was changed to VVI 60 bpm with underlying rhythm of atrial fibrillation with ventricular rate of 40–80 bpm.

### Patient 2

A 29-year-old man with a history of intravenous drug abuse and prior methicillin-resistant *Staphylococcus aureus* tricuspid valve endocarditis requiring angiovac debulking presented with *Streptococcus mitis* tricuspid valve endocarditis and septic pulmonary emboli. He was undergoing tricuspid valve replacement via sternotomy and was placed on cardiopulmonary bypass via the right femoral vein and the distal ascending aorta. When a right atriotomy was performed, the septal leaflet of the tricuspid valve was found to be degraded, likely from prior angiovac procedures and endocarditis. After debridement, a series of 2-0 pledgeted annular stitches were placed with everting technique along the annulus of the tricuspid valve to properly anchor the new prosthetic valve. Despite being shallow along the septal annulus between the anteroseptal commissure and coronary sinus, the patient displayed a new-onset complete heart block. Because the tricuspid septal leaflet was degraded, and stitches needed to be placed in a true thickness of a very large annulus, the surgeon felt that the heart block would not resolve and that the best course of action was a permanent pacemaker. Electrophysiology was consulted intraoperatively and agreed that leadless pacemaker would be the best option given his infection risk and the likely pacing dependency after surgery.

The Micra delivery system was advanced to the right ventricle. The initial deployment of the Micra AV (MC1AVR1) device was unsuccessful, with the tug test revealing lack of proper tension on the tethers. The device was recaptured, and the surgeon repositioned the sheath. After deployment, the tug test showed appropriate tension on the tethers and the video showed engagement of at least 3 of the tines in the muscular trabeculations. The surgery did not involve cardioplegia and thus testing of the pacemaker was performed immediately, showing R-wave sensing of 8.9 mV, lead impedance of 1490 Ω, and pacing threshold of 1.75 V @ 0.24 ms. The tethers were cut and removed. The device was programmed to VVI 70 bpm. Afterward, the surgeon completed the tricuspid valve replacement using a 33 mm Mitris valve (Edwards, Irvine, CA). The following day, his device was interrogated, showing stable R waves of 7.3 mV, impedance of 1180 Ω, and threshold of 0.25 V @ 0.24 ms. The device was programmed to VDD at 80 bpm with AV conduction mode switch on. The patient did well and was discharged on postoperative day 7 with plans for a total of 6 weeks of intravenous antibiotics. On his 1-month remote interrogation, the leadless pacemaker showed a pacing threshold of 0.3 V @ 0.24 ms and stable device parameters with R-wave sensing of 8.3 mV and 860 Ω impedance. The patient has yet to follow up in device clinic for an in-person interrogation.

## Discussion

We present 2 cases of direct transcatheter Micra leadless pacemaker implantation at the time of the valve surgery. A novel video methodology to confirm the engagement of tines in trabeculations is described. During standard leadless pacemaker implantation, fluoroscopy and cinegraphy is used to observe flaring of the tines during tug test to confirm its engagement. Fluoroscopy was not used in our cases owing to its cumbersome nature during open heart surgery. Besides, we felt that direct visualization via the video confirmed tine engagement.

These cases illustrate the feasibility and advantage of implanting a Micra leadless pacemaker during open heart surgery if there is preoperative or intraoperative indication for permanent pacing. Direct visualization confirms the septal location of implantation, avoiding the risk of cardiac perforation. Additionally, potential vascular complications associated with venous access with large-bore catheters are avoided. There were several unique challenges during intraoperative Micra implantation that required additional consideration. First, leadless pacemaker and its design were intended to be performed via a transfemoral vein approach under fluoroscopy.^1^ Using a portable C-arm during open heart surgery is cumbersome and we did not perform fluoroscopy to confirm anatomical location of the placement or tine engagement. Instead, the RV septal location and tine engagement are directly visible intraoperatively. In Supplemental Video 1, 3 tines are seen engaged with malformation of the trabeculation by the tine tips on tug test. Second, testing of the device for sensing, impedance, and threshold immediately after implantation could not be done in case 1 owing to cardioplegia but was available in case 2. Inability to test the device until cardiac activity is restored during surgery was a limitation in the open heart surgery approach in patient 1. Back-up plans should be considered if the pacing and sensing parameters are inadequate when tested. We anticipated manually repositioning the Micra pacemaker and performing a tug test if that scenario arises. In patient 2, the patient was on cardiopulmonary bypass and then the tricuspid valve was exposed while keeping the left heart in normal cardiac motion. This allowed testing of electrical measurement of the device at the time of deployment.

Tricuspid valve replacements pose unique challenges to implantation of a standard transvenous pacemaker with the RV lead traversing the prosthetic valve. Both cases were done in patients undergoing tricuspid valve replacements, which made the choice of leadless pacemaker beneficial. Intraoperative implantation also avoids a separate procedure with its potential complications. Additionally, postoperative temporary pacing via epicardial leads is sometimes unreliable and the leadless intraoperative permanent pacing provides a more reliable pacing option, especially in pacing-dependent patients. The elevated pacing threshold in patient 1 illustrates the limitations of pacemaker implantation during cardioplegia. A deliberation about the advantages and disadvantages of intraoperative leadless pacemaker implantation should be considered among the cardiothoracic surgeon, electrophysiologist, and patient.

Intraoperative leadless pacemaker implantation has been described previously in literature, with similar challenges and limitations.^6^^,^^7^ Shivamurthy and colleagues^7^ described 15 successful cases of leadless pacemaker implantation under direct visualization during open heart valve surgery. They described the use of external tug test (using the Micra tethers) and an internal tug test (using forceps) to confirm adequate fixation to the RV myocardium. However, the study did not directly confirm engagement of tines in the trabeculations, a step we believe could add to the safety, minimizing risk of device dislodgment. When feasible, we propose the use of direct visualization of tine engagement (Figure 1 and Supplemental Video 1) as a surrogate to fluoroscopic confirmation of adequate fixation.

## Conclusion

Intraoperative leadless Micra pacemaker implantation may be considered in patients with pacing indications undergoing tricuspid valve replacement. Use of videoscope to confirm tine engagement in RV trabeculations is a novel technique that could add to the efficacy of the procedure.

## Disclosures

None.

## Appendix Supplementary Data

Supplemental Video 1 Videoscope assisted Micra^TM^ leadless pacemaker implantation technique. First the Micra^TM^ delivery sheath was advanced into the RV septum by the surgeon. The tricuspid valve, RV septum and trabeculations, and moderator band are directly visualized. Second, the Micra^TM^ pacemaker was advanced into the heart and deployed. Next, the tug test was performed which showed the three different tines engagement with malformation and retraction of the trabeculations.
